# A Small Scale Optically Pumped Fetal Magnetocardiography System

**DOI:** 10.3390/jcm12103380

**Published:** 2023-05-10

**Authors:** David Wurm, Peter Ewert, Peter Fierlinger, Ronald T. Wakai, Verena Wallner, Lena Wunderl, Annette Wacker-Gußmann

**Affiliations:** 1 Chair E66, School of Natural Sciences, Technical University of Munich, 80636 Munich, Germany; 2 Department of Congenital Heart Disease and Pediatric Cardiology, German Heart Center, 80636 Munich, Germany; 3 Department of Medical Physics, University of Wisconsin Madison, Madison, WI 53706, USA

**Keywords:** fMCG, OPM, MSR, low cost, fetal arrhythmia, cardiac time intervals, person-size shields

## Abstract

Introduction: Fetal magnetocardiography (fMCG) is considered the best technique for diagnosis of fetal arrhythmia. It is superior to more widely used methods such as fetal, fetal electrocardiography, and cardiotocography for evaluation of fetal rhythm. The combination of fMCG and fetal echocardiography can provide a more comprehensive evaluation of fetal cardiac rhythm and function than is currently possible. In this study, we demonstrate a practical fMCG system based on optically pumped magnetometers (OPMs). Methods: Seven pregnant women with uncomplicated pregnancies underwent fMCG at 26–36 weeks’ gestation. The recordings were made using an OPM-based fMCG system and a person-sized magnetic shield. The shield is much smaller than a shielded room and provides easy access with a large opening that allows the pregnant woman to lie comfortably in a prone position. Results: The data show no significant loss of quality compared to data acquired in a shielded room. Measurements of standard cardiac time intervals yielded the following results: PR = 104 ± 6 ms, QRS = 52.6 ± 1.5 ms, and QTc = 387 ± 19 ms. These results are compatible with those from prior studies performed using superconducting quantum interference device (SQUID) fMCG systems. Conclusions: To our knowledge, this is the first European fMCG device with OPM technology commissioned for basic research in a pediatric cardiology unit. We demonstrated a patient-friendly, comfortable, and open fMCG system. The data yielded consistent cardiac intervals, measured from time-averaged waveforms, compatible with published SQUID and OPM data. This is an important step toward making the method widely accessible.

## 1. Introduction

The introduction of ultrasound and echocardiography in fetal evaluation significantly improved the diagnosis and treatment of fetal cardiac disease, leading to a major reduction in prenatal morbidity and mortality. Currently, however, conventional methods are limited in their ability to diagnose cardiac arrhythmia in the fetus. This is due to the fact that assessment of fetal rhythm by fetal echocardiography is indirect. To address this problem, many groups have attempted to develop fetal electrocardiography; however, it is still not used clinically despite many years of research. The electrically insulating effect of the vernix, which develops from the 17th week of pregnancy onwards, remains problematic for fetal electrocardiography. Cardiotocography (CTG) devices are commonly used in routine obstetric care but are based on Doppler methods and provide heart rate but not cardiac intervals.

Several studies sought to use CTG to assess heart rate variability [1] and fetal development [2,3]. However, the use of CTGs remains limited to assessing normal fetal rhythm and heart rate in the third trimester and during labor, leaving a large gap in the care of high-risk pregnancies in the second trimester. Some groups sought to address this gap by developing fetal magnetocardiography [4,5] (fMCG). FMCG can diagnose fetal arrhythmias more precisely than echocardiography. It allows extended Holter-like monitoring, and depolarization and repolarization times can be determined. Several studies highlighted the potential clinical use of fetal magnetocardiography in fetuses at high risk for arrhythmia. fMCG added significant new findings in addition to the referral echocardiogram, and in almost half of the patients, it changed clinical management [6,7,8]. Especially pregnancies with fetal inherited arrhythmia syndromes such as long QT syndromes require exceptional preparedness and a high level of understanding to optimize precise fetal care [9]. Fetal symptoms are sometimes not detected by echocardiography, and when available, fetal MCG can be very useful in risk-stratifying these pregnancies [10,11,12,13].

Superconducting quantum interference devices [14] (SQUIDs) were the first devices to provide such tracings. Because of high installation costs (e.g., shielded rooms) and expensive consumables (e.g., liquid helium), these devices have not gained acceptance in routine fetal care. Recent advances in optically pumped magnetometers [15] (OPMs) have led to the development of compact, less expensive sensors [16,17] which can be used in person-sized, magnetic shields rather than a bulky magnetically shielded room.

Compared to ECG, the fMCG has favorable transmission properties, which allows the signal to pass through the fetal and maternal tissues with less attenuation. This enables detection of the fetal magnetic signal outside the maternal abdomen, making the method non-invasive. OPMs are passive recording devices that do not emit magnetic fields or energy, making them safe for fetal evaluation.

Here, we present the initial results of a newly built fMCG system using a comfortable, person-sized shield and a 16-channel, optically pumped magnetometer array. Measurements with this device were analyzed and compared well with existing SQUID fMCG data.

## 2. Material and Methods

The study was performed by the Chair of Precision Measurements at Extreme Condi-tions at the School of Natural Sciences and the Department of Congenital Heart Disease, German Heart Center at the Technical University Munich, Germany. 

**Figure 1:**
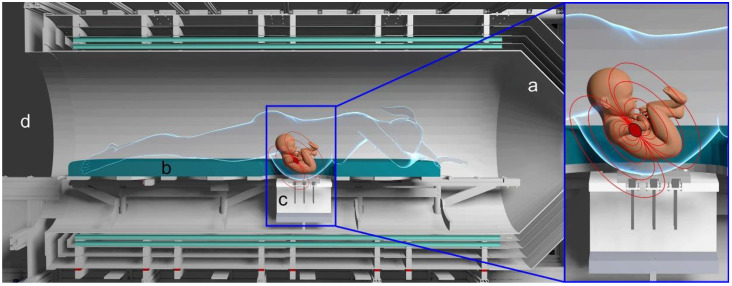
Rendering of the magnetic shielding (**a**) surrounding the bed (**b**) where the patient lies in a prone position directly above the OPM sensor support ((**c**) and insert). Idealized magnetic field lines are shown in red. The large opening grants easy access and continuous visual monitoring during measurements (**d**).

Our magnetic shield (Figure 1) consists of a cylindrical, person-sized magnetic shield built from three layers of 1.5 mm thick MuMetal^®^ by Magnetic Shields Limited, Kent, UK, (https://magneticshields.co.uk/technical/material-technical-data) and provides a noise floor of 220 fT/ Hz^1/2^ (femto Tesla (fT), 1fT = 1 × 10^−12^ tesla) per square root of Hertz expresses a the noise floor in magnetic flux density per bandwidth as a linear spectral density) in the frequency band of interest (3–45 Hz). The accessible interior, covered by a polyvinylchloride lining, has a diameter of 97 cm and length of 265 cm and is closed off on one end with a conical end cap. A static current loop at the opening provides compensation against static magnetic field influx. The tri-axial Magnetic Field Cancelling System MR-3 by Stefan Mayer Instruments Dinslaken, Germany, provides additional active field compensation, yielding an ambient noise floor of 80 fT/Hz^1/2^ (This noise floor is about 6 times larger than the magnetometer noise floor).

After changing into non-magnetic clothing, the patients lie in a prone position on a movable bed with a cutout for the abdomen. A brief ultrasound exam is performed so that the sensor array can be placed proximal to the fetal heart. The dominant noise arises from mechanical vibration from respiratory and other patient movements, which couple to the inner shield layers. Vibrations with an amplitude of 0.1 mm generate artifacts with a magnitude of several thousand femtoteslas and visually obscure the fetal heart signal in the raw tracings.

The set of eight QuSpin Zero Field Magnetometers (QZFMs) from QuSpin Inc., Louisville, CO, USA, are fixed in a flat 3 × 3 grid with 5 cm grid spacing below the abdomen. Shimming allows adjustment for differences in abdomen size. In this configuration, the surface of the abdomen is 1.5 mm above the sensor’s surface or 8 mm above the sensitive volume of the magnetometer. To mitigate the thermal irritation from the sensor, a thin layer of air passes between the sensor head and the top surface, which isolates both sides and removes excess heat.

The OPM sensors are operated in dual-axis mode. An analog-to-digital converter with millisecond resolution digitizes the sensor outputs. Each measurement consists of a ten-minute continuous recording. In general, there is no limit on the number or duration of the measurements, similar to Holter or ICU monitoring.

A typical session consists of two to three measurements with modest variation in the position of the abdomen to allow different waveform configurations to be recorded. The raw signal is displayed in real-time to monitor the quality of recorded data. In postprocessing, the signal id passed through a software 50 Hz notch filter to remove power line noise and a first-order Butterworth filter with 3–75 Hz passband.

Independent component analysis (FastICA [18]) is used to isolate the fetal and maternal signal components. For these components, we apply bispectral analysis to perform matched filtering [19]. From the output of this filter, we detect the occurrence of heart beats, from which the instantaneous heart rate is computed. High-resolution fMCG waveforms are derived by averaging up to 300 consecutive heart beats. A multi-channel overlay of the averaged waveforms is used to measure the following waveform intervals: P-wave duration, PR interval, QRS interval, and QT interval.

## 3. Results

### 3.1. Patient Characteristics

Measurements were conducted in healthy pregnant women with uncomplicated pregnancies. The mean age of the mothers was 30.5 ± 2.1 years, and they were studied at 32.4 ± 3.4 weeks’ gestation. One pregnant woman had cardiolipin antibodies and was on aspirin (100 mg). All other pregnant women had no family history of cardiovascular or other diseases and had no medication intake.

### 3.2. Patient Comfort

All pregnant women were queried about their well-being during the procedure, and each reported a high level of comfort. We attribute this to the shield’s wide opening and the quiet ambiance. Several women fell asleep during sessions due to the relaxing post.

### 3.3. Fetal Cardiac Time Intervals

**Figure 2:**
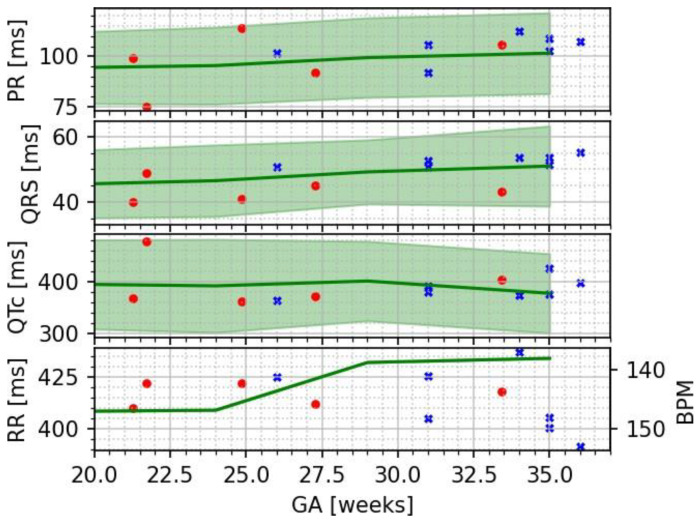
Plot of the cardiac time intervals (crosses) shown along reference data adapted from [20] (circles) and SQUID data adapted from [21] as solid lines with shaded bands spanning from the 5th to the 95th percentile.

Resolution of all fMCG waveform components was confirmed. The intervals were compatible with both SQUID-based and OPM-based fMCG studies published by Wakai R. T. et al. [20,21], which serve as a database of normative values for investigation of fetal arrhythmias with fMCG technology (See Figure 2).

Most fetal cardiac time intervals show a positive correlation with gestational age (GA); however, the ST segment, T-wave duration, and QT interval did not. These trends are consistent with prior fMCG studies of fetal heart development during pregnancy (Table 1).

## 4. Discussion

Our results are in good agreement with those from prior OPM-based studies of normal fetuses. As it was previously shown that SQUID- and OPM-based fMCG data are equivalent [20], we could use normative values from large SQUID data sets to confirm that our data are within expected ranges (Figure 2). Future work will include comparative OPM/SQUID measurements in individual pregnancies to improve statistical comparisons. Our data also show that the PR and QRS intervals increase with gestational age, in agreement with prior published studies [20,21,22].

While the cardiac intervals in this work were measured from waveforms obtained by averaging of up to 300 beats, in two subjects the signal components could be resolved without averaging. To achieve this consistently is an important future goal. From a technical standpoint, the most challenging aspect of our study was the use of an open-ended magnetic shield, which reduced anxiety and the possibility of claustrophobia but allowed environmental magnetic interference to enter the shield. In addition to direct coupling of interfering magnetic fields, large magnetic field gradients across the sensors make them susceptible to vibration and movement artifact. The typical frequency of vibrations is several Hz, and this artifact can obscure low-frequency features like the T-wave. Ongoing development of the shield will target improved active compensation of gradients around the sensors and mechanical measures to dampen vibration.

Early diagnosis is important for clinical application. Fetal cardiac time intervals of early pregnancies are in general more challenging due to smaller signal strength. Our current IRB protocol only allows the study of pregnancies after 25 weeks’ gestation. The potential of our fMCG system for use in early pregnancy remains to be demonstrated and is the subject of ongoing work.

## 5. Conclusions

We demonstrated a patient-friendly, comfortable, open fMCG system based on OPM technology. The data yielded consistent cardiac intervals, measured from time-averaged waveforms, compatible with published SQUID and OPM data. Unaveraged signal analysis will require further technical improvements. Our OPM-based fMCG system shows excellent potential for use in fetal cardiac evaluation.

## Figures and Tables

**Table 1:** Interval length for PR, QRS, and QTc (based on Bazett’s formula) for seven measurements. Ranges represent a standard deviation within this sample or, in the case of RR-length, the variation of the underlying recording.

	GA [Weeks]	PR [ms]	QRS [ms]	QTc [ms]	RR ± SD [ms]
1	35	102	53	427	406 ± 7
2	31	92	53	391	405 ± 10
3	35	109	52	375	400 ± 17
4	26	102	51	364	425 ± 13
5	34	113	53	374	437 ± 11
6	36	107	55	397	392 ± 18
7	31	106	51	379	425 ± 13
Ø *±* SD	32.6 ± 3.2	104 ± 6	52.6 ± 1.5	387 ± 19	413 ± 15

## Data Availability

Data is available on reasonable request.
